# Vaccine-induced Human Antibodies Specific for the Third Variable Region of HIV-1 gp120 Impose Immune Pressure on Infecting Viruses

**DOI:** 10.1016/j.ebiom.2014.10.022

**Published:** 2014-11-05

**Authors:** Susan Zolla-Pazner, Paul T. Edlefsen, Morgane Rolland, Xiang-Peng Kong, Allan deCamp, Raphael Gottardo, Constance Williams, Sodsai Tovanabutra, Sandra Sharpe-Cohen, James I. Mullins, Mark S. deSouza, Nicos Karasavvas, Sorachai Nitayaphan, Supachai Rerks-Ngarm, Punnee Pitisuttihum, Jaranit Kaewkungwal, Robert J. O'Connell, Merlin L. Robb, Nelson L. Michael, Jerome H. Kim, Peter Gilbert

**Affiliations:** a New York Veterans Affairs Harbor Healthcare System, 423 East 23rd Street, New York, NY 10010, USA; b New York University School of Medicine, 550 First Avenue, New York, NY 10016, USA; c Vaccine and Infectious Disease Division, Public Health Sciences Division, Fred Hutchinson Cancer Research Center, 1100 Fairview Ave. N., M2-C200, Seattle, WA 98109, USA; d Department of Retrovirology, Walter Reed Army Institute of Research, Building 503, Silver Spring, MD 20910, USA; e Department of Microbiology, University of Washington, 358B Rosen Building, Campus Box 358070, Seattle, WA 98195, USA; f Thai Red Cross AIDS Research Center 104, Tower 2, Rajdamri Rd., Pathumwan, Bangkok 10330, Thailand; g Armed Forces Research Institute of Medical Science (AFRIMS) Department of Retrovirology, Humoral Immunology and Assessment Laboratory, 315/6 Rajvithi Rd., Bangkok 10400, Thailand; h Department of Disease Control, Ministry of Public Health, Nonthaburi 11000, Thailand; i Department of Clinical Tropical Medicine, Mahidol University, 420/6 Ratchawithi Road, Ratchathewi, Bangkok 10400, Thailand; j U.S. Army Military HIV Research Program, 6720A Rockledge Dr., Suite 400, Bethesda, MD 20817, USA

**Keywords:** HIV, Antibody, Vaccine, Clinical trial

## Abstract

To evaluate the role of V3-specific IgG antibodies (Abs) in the RV144 clinical HIV vaccine trial, which reduced HIV-1 infection by 31.2%, the anti-V3 Ab response was assessed. Vaccinees' V3 Abs were highly cross-reactive with cyclic V3 peptides (cV3s) from diverse virus subtypes. Sieve analysis of CRF01_AE breakthrough viruses from 43 vaccine- and 66 placebo-recipients demonstrated an estimated vaccine efficacy of 85% against viruses with amino acids mismatching the vaccine at V3 site 317 (p = 0.004) and 52% against viruses matching the vaccine at V3 site 307 (p = 0.004). This analysis was supported by data showing that vaccinees' plasma Abs were less reactive with I^307^ when replaced with residues found more often in vaccinees' breakthrough viruses. Simultaneously, viruses with mutations at F^317^ were less infectious, possibly due to the contribution of F^317^ to optimal formation of the V3 hydrophobic core. These data suggest that RV144-induced V3-specific Abs imposed immune pressure on infecting viruses and inform efforts to design an HIV vaccine.

## Highlights

• The RV144 vaccine reduced infection by viruses with isoleucine in V3 position 307.
• Many vaccine-induced antibodies are cross-reactive and target an epitope including I^307^.
• There was selection for breakthrough viruses carrying F^317^ in V3 (p = 0.004).
• F^317^ is needed to maintain optimal infectivity.
• F^317^ is a poor or non-contact residue for vaccine induced V3 antibodies.

## 1 Introduction

The vaccine tested in the RV144 Thai clinical trial provided modest protection in healthy heterosexual individuals against HIV-1 infection. To date this is the only HIV clinical trial to demonstrate vaccine efficacy (VE), albeit at a level of 31.2% (Rerks-Ngarm et al., 2009). A case–control study designed to identify immune correlates of reduced infection risk demonstrated that high levels of antibodies (Abs) directed against the V1V2 region of the virus gp120 envelope glycoprotein were associated with a decreased risk of HIV-1 infection, while high levels of Env-specific plasma IgA were associated with an increased risk (Haynes et al., 2012). The V1V2 vaccine-induced Abs were shown to be broadly reactive with the V2 region of multiple subtypes despite immunization with immunogens from only subtypes B and CRF01_AE (Zolla-Pazner et al., 2013, Zolla-Pazner et al., 2014). Genetic studies that compared the V1V2 region of viruses infecting placebo and vaccine recipients identified two positions in V2, 169 and 181, that distinguished viruses from vaccine recipients, resulting in VEs of 48% and 78%, respectively (Rolland et al., 2012). This sieve analysis complemented the finding of an association between high V1V2-binding Abs and reduced HIV-1 acquisition, and provided independent evidence that vaccine-induced V2 Ab responses plausibly had a role in the reduced rate of infection associated with the RV144 vaccine regimen (Liao et al., 2013, Liu et al., 2013, Zolla-Pazner et al., 2013, Pollara et al., 2014, Yates et al., 2014).

Additional analyses showed that plasma Abs from RV144 vaccinees reactive in a microarray to a linear V3 peptide from CRF01_AE were also an inverse correlate of infection risk, but only in vaccine recipients who had lower levels of Env-specific plasma IgA and aggregate neutralizing Ab activity (Gottardo et al., 2013), and V3-specific Abs from RV144 vaccinees were shown to capture infectious virions, including the vaccine strain CM244 (Liu et al., 2013). These and previous data (Karasavvas et al., 2012) generated the hypothesis that IgG Abs to epitopes in both the V2 and V3 regions of gp120 are part of a complex interplay of immune responses that contributed to the reduced rate of infection in RV144 participants, and suggested that further delineation of additional elements of the V3 Ab response might reveal important information about the factors involved in blocking HIV infection.

Extensive data in the literature support a possible protective role for Abs directed against the crown of the V3 loop. The proof of principle that human monoclonal Abs (mAbs) specific for the V3 loop could react with and neutralize many strains of HIV, including lab-adapted and primary isolates from several HIV subtypes, was established 20 years ago (Gorny et al., 1992, Gorny et al., 1993, Conley et al., 1994). Cross-clade neutralization by V3 Abs has now been confirmed (Gorny et al., 1997, Gorny et al., 2002, Corti et al., 2010, Hioe et al., 2010, Mouquet et al., 2011, Klein et al., 2012), and V3 mAbs have been shown to provide sterilizing immunity by passive immunization in various animal models (Emini et al., 1992, Safrit et al., 1993, Andrus et al., 1998, Eda et al., 2006, Watkins et al., 2011). Furthermore, the V3 region is one of the most immunogenic epitopes in the envelope, inducing glycan-independent Abs directed at the crown of the V3 loop in > 90% of HIV-infected individuals (Gorny et al., 1997, Gorny et al., 2002, Zolla-Pazner, 2005). These Abs show cross-clade immunochemical and neutralizing activity (Gorny et al., 2004, Corti et al., 2010, Hioe et al., 2010, Mouquet et al., 2011) despite the fact that their CDR H3 regions are comparable to the average length of this segment (mean of 16 amino acids), and their VH regions differ from germline by a mean of only 8.6% (Andrabi et al., 2013). A second type of V3-directed Ab is glycan-dependent, specific for the base of V3 and the adjacent glycan in C4; these Abs are extremely broadly reactive and potent, but are induced in only a small percentage of infected individuals, and their heavy chains are extensively mutated from germline (27–34%), reflective of somatic hypermutation which requires several years of exposure to antigen (Pejchal et al., 2011, Walker et al., 2011, Mascola and Haynes, 2013).

An early indication that V3 bears conserved structural elements allowing the broad cross-reactivity of V3 Abs directed to the crown of the V3 loop came from data showing that V3 participates in the binding of gp120 to the cell surface coreceptors CCR5 or CXCR4 and in determining virus tropism (Shioda et al., 1991, Shioda et al., 1992, Trkola et al., 1996, Hill et al., 1997). Subsequently, extensive crystallographic studies of V3 mAbs in complex with V3 peptides revealed a generic β-strand/turn/β-strand structure for the V3 crown encompassing a hydrophobic core (often composed of the highly conserved amino acids I^307^, I^309^ and F^317^) and a hydrophilic face (including amino acids at positions 306, 308 and 316 which are positions displaying considerable variation) (Almond et al., 2010, Jiang et al., 2010, Zolla-Pazner and Cardozo, 2010).

In order to examine the V3 Abs induced by the RV144 vaccine regimen, the immunochemical characteristics of V3-specific Abs in vaccine recipients were studied. Simultaneously, V3 sequence variation in viruses infecting vaccine and placebo recipients were compared and key V3 residues were analyzed to understand their contribution to optimal V3 structure and function. The results of these analyses complement one another, suggesting that V3 Abs played a role in reducing the infection rate and exerting immune pressure on the breakthrough viruses by selecting *against* viruses with the consensus Ile at position 307 and selecting *for* viruses that retain Phe at position 317.

## 2 Materials and Methods

### 2.1 Ethics Statement

The RV144 clinical vaccine trial was registered with ClincialTrials.gov and assigned a registration number of NCT00223080. It was approved by all relevant institutional and governmental committees, and the protocol of the trial was described in (Rerks-Ngarm et al., 2009). Specifically, the institutional review boards of the Thai Ministry of Public Health Ethics Committee, the Royal Thai Army Medical Department, Ethics Committee of the Faculty of Tropical Medicine, Mahidol University, and the US Surgeon General's Human Subjects Research Review Board approved the protocol and attendant immune correlates work. All subjects provided written informed consent and passed a test of understanding as previously described (Rerks-Ngarm et al., 2009). Briefly, RV144 was a community-based, randomized, multicenter, double-blind, placebo-controlled vaccine efficacy trial consisting of four injections of a recombinant canarypox vector vaccine (ALVAC-HIV [vCP1521]) given at 0, 1, 3, and 6 months, and two injections of recombinant gp120 subunits (AIDSVAX B/E®) given at months 3 and 6. The vaccine and placebo injections were administered to 16,402 healthy men and women between the ages of 18 and 30 years in Thailand.

### 2.2 Plasma and Peptides Used

The plasma set used here consisted of plasma from 20 placebo recipients and 40 vaccine recipients participating in the RV144 trial who were HIV seronegative at the end of the study period; blood specimens had been collected at weeks 0, 26 and 52.

Cyclic V3 peptides (cV3) were synthesized commercially (Biopeptide Co., San Diego, CA) using the sequences of viruses from subtypes A, B, C, CRF01_AE and CRF02_AG (Table 1). These peptides had a biotin residue and a three glycine linker covalently bound to the N-terminal cysteine. In addition, a linear V3 peptide, biotin-GGGSNNTRTSITIGPGQVFYRTGD, representing the consensus sequence of CRF01_AE was synthesized (Biopeptide Co.) since IgG to a linear peptide with this V3 sequence tested by microarray significantly inversely correlated with infection risk (OR = 0.54 per SD increase, p = 0.0042) (Gottardo et al., 2013).

### 2.3 ELISA Assay

StreptaWell plates (Roche) were coated with 1 μg/ml biotinylated cV3s for 1.5 h at 37°C and then washed six times with phosphate-buffered saline containing 0.05% Tween-20, pH 7.4, before incubation for 1.5 h at 37 °C with RV144 plasma diluted 1:100 in RPMI medium containing 15% fetal bovine serum. The plates were washed six times, and alkaline phosphatase-conjugated goat anti-human IgG (1:2000) was added for 1.5 h at 37 °C. After washing, 10% diethanolamine substrate was added for 30 min to develop color, and the plates were read at Å405 nm. At each step, every well contained 50 μl; specimens were run in duplicate in each experiment, and two or three experiments were performed with each plasma/peptide combination.

### 2.4 Site-directed Mutagenesis, Production of Pseudoviruses in 293T Cells, and Infectivity Assays

Point mutations were introduced in the V3 region of plasmids TH023.06 and CM244OR.01 which were kindly supplied by Dr. Agnes-Laurence Chenine at the H. M. Jackson Foundation using the QuikChange II XL Site-Directed Mutagenesis Kit (Stratagene, La Jolla, CA, USA), according to the manufacturer's instructions. All mutant constructs were sequenced to confirm the correct amino acid change. Wild type or mutant plasmids were used to co-transfect 293T human embryonic kidney cells (ATCC, Manassas, VA, USA) with a pSGdelta_env backbone plasmid at optimal backbone to envelope ratios, using Fugene HD (Roche, Manheim, Germany) according to the manufacturer's protocol. After 48–72 h, supernatants containing the secreted mutant or wild type pseudoviruses were harvested, filtered, aliquoted and stored at − 80 °C. Infectivity was tested by titration of supernatants using TZM.bl indicator cells (JC53BL-13, obtained from the NIH AIDS Research and Reference Reagent Program). Briefly, five-fold serial dilutions of *env-*pseudotyped virus stocks were incubated for 48 h in quadruplicate with TZM.bl cells and assayed for *luc* reporter gene activity using Bright-Glo (Promega) under standard TZM.bl assay conditions.

### 2.5 Sequence Analysis

Viral genomes were sequenced following endpoint-dilution PCR of viral RNA from plasma specimens collected at the time of HIV-1 diagnosis (GenBank accession numbers JX446645–JX448316). Details of the sequencing of the viruses were previously described, and sequences from the 109 independent infections with CRF01_AE viruses were used in the sieve analysis (Rolland et al., 2012).

### 2.6 Sieve Analysis

Differential VE by genotype was assessed as previously described (Lunn and McNeil, 1995, Gilbert, 2000) using one representative sequence per individual, i.e., the HIV-1 genomic sequence that is the closest to the consensus sequence derived from all the sequences obtained from that individual (Rolland et al., 2012).

### 2.7 Covariation

Associations between residues were assessed using the Kullback–Leibler divergence covariation and differential covariation tests (Gilbert et al., 2005) and two Bayesian graphical methods were used which are phylogenetic comparative methods that explicitly model the evolutionary history of the sequences (Poon et al., 2007, Carlson et al., 2008).

### 2.8 Vaccine Efficacy

Genotype-specific VE was assessed with the Cox proportional hazards model and score test as described by Prentice et al. (1978), and genotype-specific VE was calculated based on one representative sequence per individual. Negative VE values are shown in symmetrized form (as the negative of the VE value calculated with vaccine and placebo groups interchanged).

### 2.9 Statistical Analyses

The mean difference between pairs of cV3 antigens was tested using a paired t-test of the null hypothesis that the true mean difference is zero. Adjusted P-values are based on the Holm FWER adjustment procedure (Holm, 1979).

### 2.10 Funding Sources

This work was supported in part by contracts between the NYU School of Medicine and the Narrows Institute of the VA Harbor Healthcare System with the Military HIV Research Program (Contracts 692526 and 793356), through funds from the Department of Veterans Affairs, Veterans Health Administration, and by grants from the National Institutes of Health (P01 AI100151 [SZP and XPK] and R01 HL59725 [SZP]). The work was also supported by an Interagency Agreement Y1-AI-2642-12 between the U.S. Army Medical Research and Material Command (USAMRMC) and the National Institutes of Allergy and Infectious Diseases and by a cooperative agreement (W81XWH-07-2-0067) between the Henry M. Jackson Foundation for the Advancement of Military Medicine, Inc., and the U.S. Department of Defense. The content is solely the responsibility of the authors and does not necessarily represent the official views of the National Institutes of Health, the Department of Defense, or the Department of Veterans Affairs who did not participate in the interpretation of data, the decision to submit the manuscript for publication, or the writing of this paper.

## 3 Results

### 3.1 Assessment of the Cross-reactivity and Longevity of V3 Abs in Plasma of RV144 Vaccinees

A plasma set from the previously described RV144 vaccine trial (Rerks-Ngarm et al., 2009) was used for these studies. The plasma were derived from study participants and consisted of plasma from 20 placebo recipients and 40 vaccine recipients who were HIV seronegative at the end of the study period; the specimens used had been collected at weeks 0, 26 (two weeks after the last immunization) and 52.

**Fig. 1:**
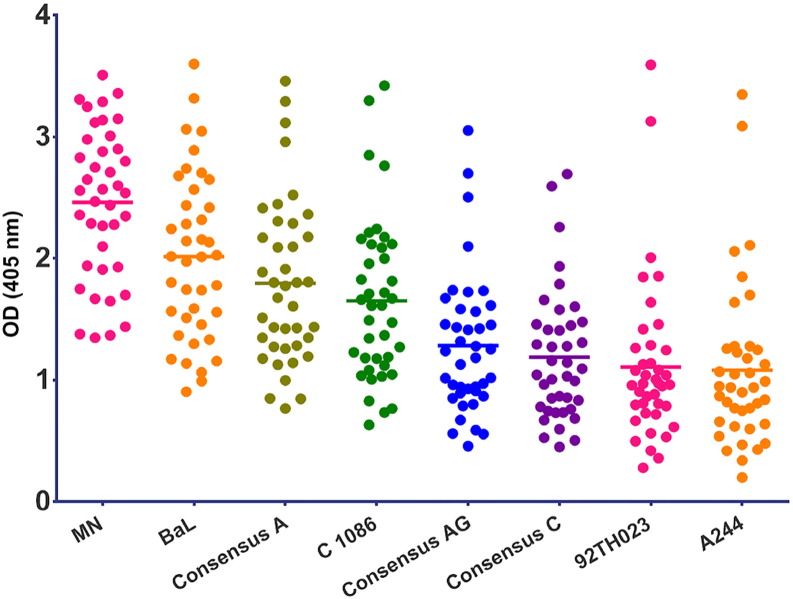
ELISA reactivity with cyclic V3 peptides of RV144 vaccinees' plasma (n = 40) drawn at week 26 (two weeks after the last immunization). The y-axis shows optical density readings at 405 nm for each plasma specimen against a given cyclic V3 peptide (identified on the x-axis). Plasma from placebo recipients were non-reactive and are not shown. All the cyclic V3 peptides were tested in two to three experiments, with two replicates in each experiment; a representative experiment is shown. As shown in Table S1, each group was significantly different from one another (Holm adjusted p-values less than 0.05) except for Conc C vs. A244 (p = 0.086), Conc C vs. 92TH023 (p = 0.16) and 92TH023 vs. A244 (p = 0.16).

Cyclic V3 peptides representing this region from subtypes A, B and C and from CRF01_AE (AE) and CRF02_AG (AG) (Table 1) were used to assess the cross-reactivity of V3 Abs in the plasma of the panel of RV144 vaccine and placebo recipients. All specimens were tested at a dilution of 1:100. The response rate to cV3s by vaccinees' specimens at week 26 ranged from 87.5% (35/40) for cV3_AE(92TH023)_, to 95% (38/40) for cV3_AE(244)_ and to 100% for consensus A, AG, and C, and subtype B strains MN and BaL cV3s (Table 2). Thus, like the V2 Ab response (Zolla-Pazner et al., 2013, Zolla-Pazner et al., 2014), the V3 Ab response generated by the RV144 immunization regimen was highly cross-reactive. The strongest response was detected with the cV3 peptide homologous to that of the gp120_MN_ protein boost, while the weakest responses were detected to the cV3s CRF01_AE (A244 and 92TH023) despite the fact that gp120_AE(A244)_ was also used as a gp120 protein boost (Fig. 1). The data were also analyzed to determine how well the responses to various cV3s correlated with one another. These data, shown in Supplementary data Fig. S1, indicate that Spearman correlation coefficients range from 0.48 to 0.98 for the wild type cV3s. For example, a correlation of 0.80 was achieved for reactivity with cV3_92TH02_ and cV3_Consensus A_ and a correlation of 0.94 was achieved for cV3_BaL_ and cV3_Consensus A_ indicating that the strength of responses correlated across clades. These data, generated with cV3s, confirm previously reported microarray data with linear V3 peptides (Gottardo et al., 2013) and indicate that the V3-specific Abs were preferentially induced by gp120_MN_. There were statistically significant differences in reactivities between 25 of the 28 pairs of the cV3 peptides when the results with the week 26 plasma were analyzed (Table S1).

To determine the longevity of the V3 Ab response, V3 Ab reactivity to the same eight cV3s was measured with plasma drawn at week 52. The reactivity decreased markedly over time in all specimens from vaccinees, but notably, many specimens continued to show positive reactivity 28 weeks after the last boost. The highest response rate in week 52 specimens when tested at a dilution of 1:100 was 55% (22/40) vs. cV3_B(MN)_ compared to 100% at week 26, while only 7.5% (3/40) of specimens from week 52 remained positive vs. cV3_AE(92TH023)_ compared to 87.5% at week 26 (Table 2).

### 3.2 Sieve Analysis of Viruses Infecting Placebo and Vaccine Recipients

A comprehensive sieve analysis of breakthrough infections in RV144 has been performed to test for evidence of vaccine-induced immune pressure across the entire HIV-1 proteome (Edlefsen et al., in press) as opposed to the original sieve analysis which only interrogated V1V2 sites (Rolland et al., 2012). In the former Edlefsen paper, site scanning methods tested all variable positions across the entire HIV-1 proteome to identify sites where VE was significantly different (p < 0.05) against two genotypes of HIV-1 defined by match or mismatch of the amino acid (AA) to the AA at that site in the vaccine; the data relevant to V3 position 317, described here, are part of this analysis in that the differential VE at site 317 was considered significant (p = 0.04). The data pertaining to V3 position 307 was not included in the Edlefsen paper since the p-value of the differential VE at site 307 was 0.065.

**Fig. 2:**
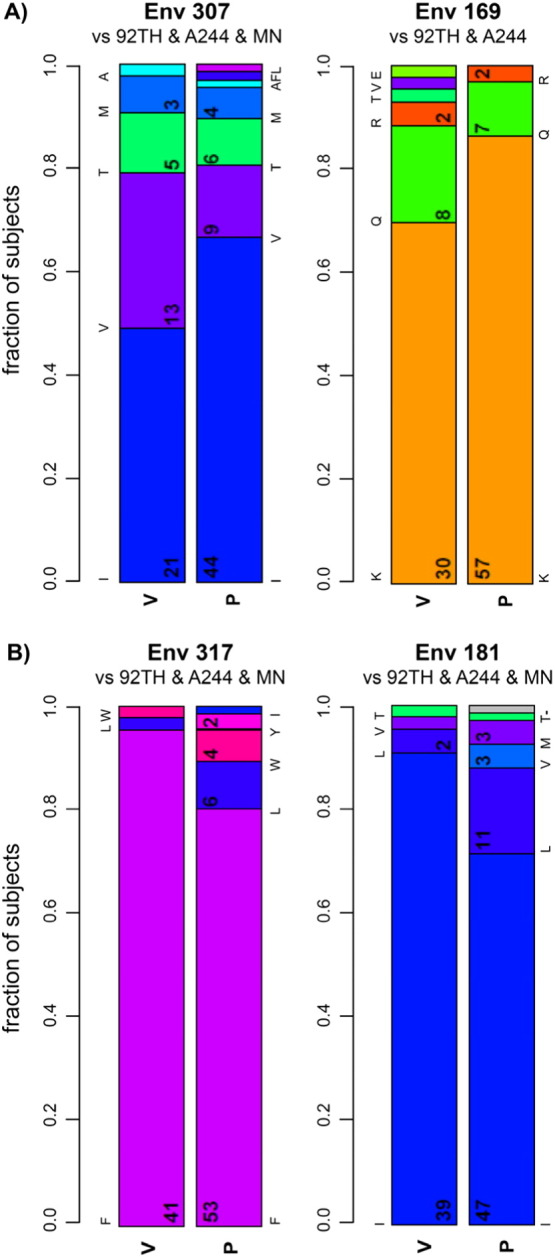
Fraction of viruses from vaccinees and placebo recipients with designated residues at V3 position 307 and V2 position 169 (A), and with designated residues at V3 position 317 and V2 position 181 (B). The vaccine immunogen amino acid is represented by the bottom-most bar in each plot. For all positions except Env 169, the amino acid was the same in all three immunogen sequences; at Env 169 the MN immunogen AA (methionine) was not found in any subject's breakthrough sequence so it is not depicted.

The consensus residues among the viruses from both the 43 infected vaccinees and 66 infected placebo recipients were isoleucine at position 307 (I^307^), and phenylalanine at position 317 (F^317^) (Fig. 2). Site 307 was *more frequently mutated* in sequences from vaccine recipients — in 51% of vaccinees compared to 33% of placebo recipients. In contrast, site 317 was *more conserved* in sequences from vaccine recipients — in 95% of vaccinees compared to 71% of placebo recipients. We verified that the vaccine status was associated with mutations at site 317 independent of the tree topology (p = 0.022), and similarly for the I307X signature (p = 0.053) (data not shown). A covariation analysis was performed to assess the relationship between sites 307 and 317; statistical significance was not reached for linkage between sites 307 and 317.

**Fig. 3:**
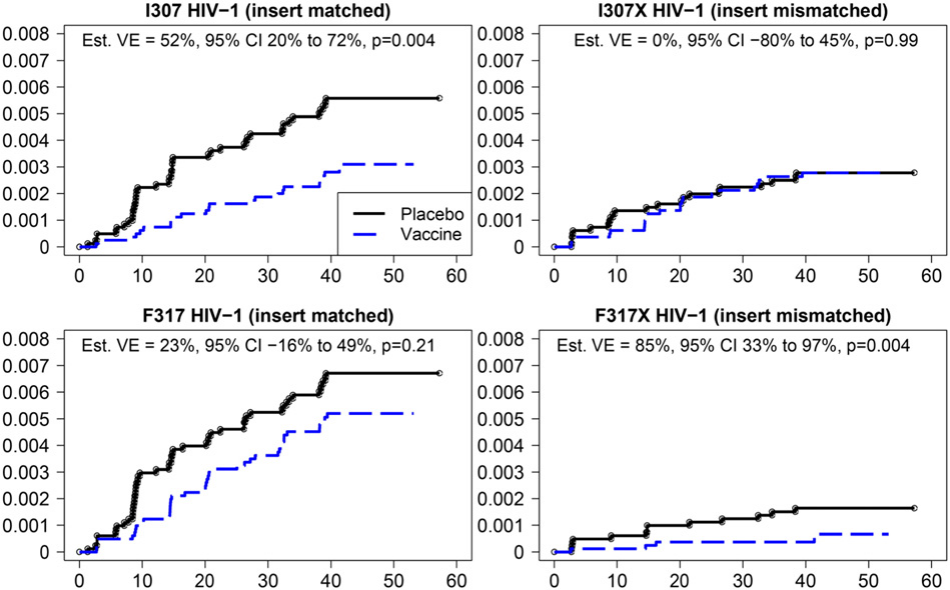
Estimated cumulative incidences of HIV-1 infection in placebo and vaccine recipients. The probability of acquiring HIV infection (y-axis) is shown for vaccine and placebo recipients whose viruses contained residues at V3 positions 307 (upper panels) or 317 (lower panels) which were either matched (I^307^ or F^317^; left panels) or mismatched (I307X or F317X; right panels) to the residues at these positions in the vaccine. The x-axis shows months since entry into the study.

Overall, VE was estimated at 31% in the RV144 trial; here we looked at VE as a function of the HIV-1 genotype at sites 307 and 317. Vaccine efficacy (VE) was increased to 52% (CI: 20–72%, p = 0.004) against viruses with I^307^, i.e., matched with the residue found at this site in the vaccine (Fig. 3, upper left and Table S2); there was no significant vaccine effect with viruses that were mismatched at position 307, i.e., I307X (VE = 0%, p = 0.99, Fig. 3, upper right and Table S2); this corresponds to a typical sieve effect. In contrast, site 317 showed an *atypical* sieve effect with a VE of 85% (CI: 32–97%, p = 0.004) against viruses with mutations at site 317 (F317X) (Fig. 3, lower right and Table S2); there was no significant VE for viruses that were matched to the vaccine at site 317 (F^317^) (VE = 23%, p = 0.21, Fig. 3 lower left and Table S2).

In addition, we analyzed differential VE for these two sites by comparing hazard ratios for one genotype vs. another (Table S3). We found that the vaccine resulted in a five-fold reduction in infections by viruses carrying F317X compared to infections with viruses with F^317^ (p = 0.040); there was a borderline significant two-fold reduction for viruses with I307X vs. I^307^ (p = 0.065). When viruses presenting both I307X and F^317^ were compared to all other genotypes, the vaccine decreased the rate of infection 3.56-fold against all other viruses (p = 0.003) (Table S3).

### 3.3 Effect of 307 Substitutions on Serum Reactivity

Recently, peptide microarray studies of plasma from RV144 vaccine recipients identified the crown of the V3 loop (^304^RTSINI..GPGQVFYRT^320^) as a highly immunogenic region and showed that when vaccine recipients had low gp120-specific plasma IgA, high responses to this linear peptide were associated with a decreased risk of HIV infection (OR = 0.49, p = 0.007) (Gottardo et al., 2013). Furthermore, while neutralization by vaccinees' specimens was weak, demonstrable neutralizing activity was due primarily to V3-specific Abs (Montefiori et al., 2012). To determine if the V3 sieve analysis and these previous studies were consistent with the immunochemical characteristics of V3 Abs in vaccinees' plasma, specimens from the plasma set described in the Materials and Methods section were analyzed for their relative reactivities with cV3s carrying substitutions at position 307 (Table 1), a primary contact residue for highly cross-reactive V3 Abs (Almond et al., 2010, Jiang et al., 2010). It has been shown that human V3 Ab responses preferentially use the VH5-51 germline gene and that these V3 Abs bind the N-terminal side of the V3 crown, with I^307^ as a key contact residue (Gorny et al., 2009).

**Fig. 4:**
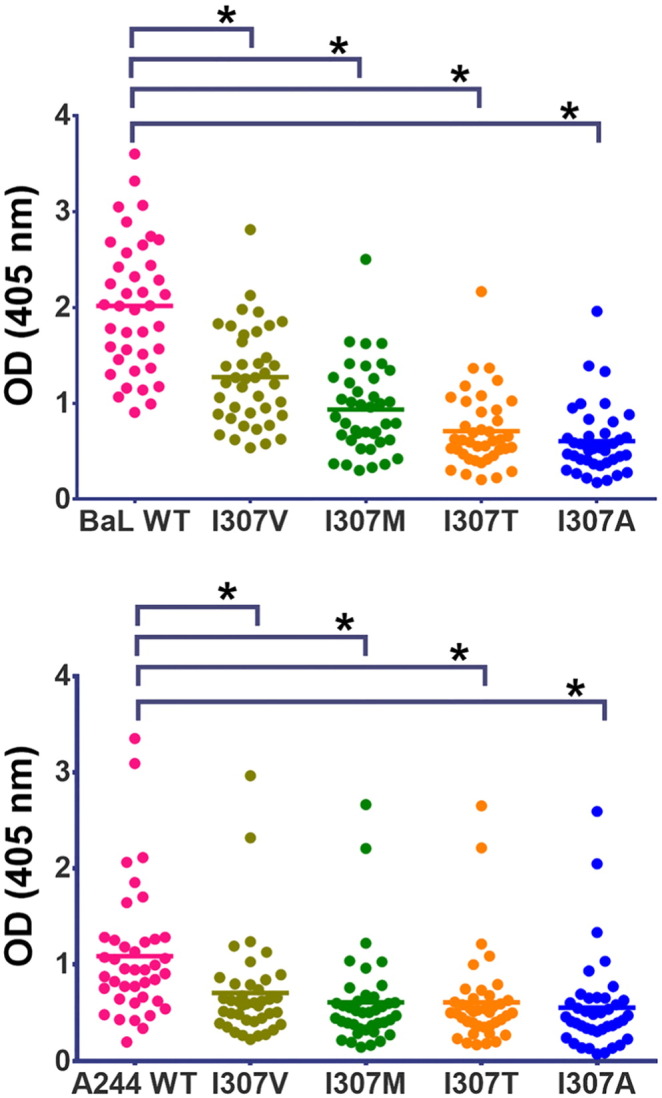
Reactivity of plasma from RV144 vaccinees (week 26) with wild type cyclic V3 peptides from clade B (strain BaL: upper panel) and clade AE (strain A244: lower panel) compared to variants with amino acid substitutions at position 307. All cyclic V3 peptides were tested in two to three experiments, with two replicates in each experiment. Asterisks denote p < 0.0001 by paired t-test.

When plasma reactivity tested by ELISA was compared between wild type (WT) cV3_B(BaL)_ and variants where I^307^ was replaced with residues found more frequently in vaccinees' breakthrough viruses than in placebo recipients (I307V, I307T, I307M, and I307A), Ab reactivity with the variants was significantly lower (p < 0.001 by paired t-test for each variant compared to WT cV3_B(BaL)_) (Fig. 4, upper panel). Similarly, when I^307^ was replaced in cV3_AE(244)_ with the same four substitutions, plasma reactivity was again significantly lower in all cases (p < 0.0001) (Fig. 4, lower panel). The data suggest that RV144-induced V3 Abs recognize an epitope that includes I^307^, supporting the sieve analysis showing that there was a decrease in the proportion of breakthrough viruses in vaccinees carrying I^307^.

### 3.4 Consequences of Substitutions at Position 317 on Virus Function

Sieve analysis also showed that F^317^ was retained in a *greater* proportion of infecting breakthrough viruses in vaccinees (Fig. 2B). Notably, F^317^ is known to often form part of the hydrophobic core of V3 that contributes to the conformation of V3 but has little or no direct contact with V3 human monoclonal Abs (Stanfield et al., 2004, Stanfield et al., 2006, Burke et al., 2009, Jiang et al., 2010), thus obviating binding experiments with F317X V3 peptides variants. However, structural data suggested an explanation for the conservation of F^317^ in viruses infecting vaccinees: F^317^ could play a structural role affecting virus fitness, similar to the role played by the V2 residue I^181^ (Spurrier et al., 2014). We therefore investigated the frequency and nature of mutations at position 317 and the effect that substitutions at this site could have on infectivity. Phenylalanine is present at position 317 in 5263/6010 (88%) viruses from multiple subtypes and CRFs that were previously analyzed (Almond et al., 2010). Only two vaccinees carried breakthrough viruses with F317X; the V3 sequences of these viruses are shown in Table 3, revealing that these viruses had five and six mutations in V3 when compared to the V3 of A244 and most of these mutations are rare in circulating CRF01_AE viruses (Table 4).

**Fig. 5:**
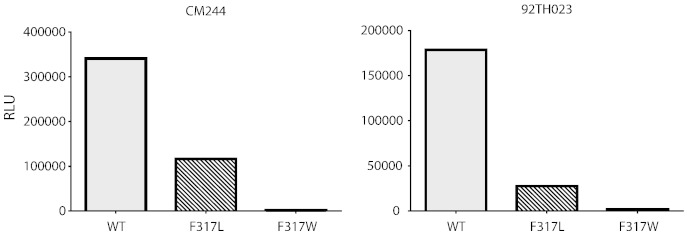
Infectivity of wild type (WT) CM244 and 92TH023 pseudoviruses and pseudoviruses with an F317L mutation or an F317W mutation.

To determine if substitutions at F^317^ affect infectivity, an F317L or an F317W mutation was made in CM244 and 92TH023. These are mutations that occur at F^317^ in the two F317X escape viruses in vaccinees (see Fig. 2B). For both CRF01_AE strains tested, substitution of F^317^ with leucine reduced infectivity by ~ 65–85%, and substitution at this site with tryptophan rendered both pseudoviruses non-infectious (Fig. 5). An F317A mutant of 92TH023, bearing another substitution that is rare but occurs at this site in nature, was also non-infectious (D. Montefiori, personal communication).

Taken together, the immunologic and genetic data suggest that vaccine-induced anti-V3 Abs exerted immune pressure on viruses bearing I^307^, *decreasing* the number of infections by viruses carrying the same I^307^ residue that RV144 V3 Abs can target (Fig. 2A). In contrast, the *increased* proportion of vaccine breakthrough viruses with F^317^ (Fig. 2B) suggests a non-immunologic mechanism for selection for viruses bearing F^317^. This selection is based on both structural and functional parameters, i.e., virus selection in the presence of Abs induced by the RV144 vaccine appears to be related to both the packing of the hydrophobic core of V3 which includes I^307^, I^309^ and F^317^ and the impact of the structure of the hydrophobic core on the accessibility of the most immunogenic V3 epitope (which includes I^307^) (Jiang et al., 2010, Xiang et al., 2010).

### 3.5 Structural Analysis of the V3 Hydrophobic Core

**Fig. 6:**
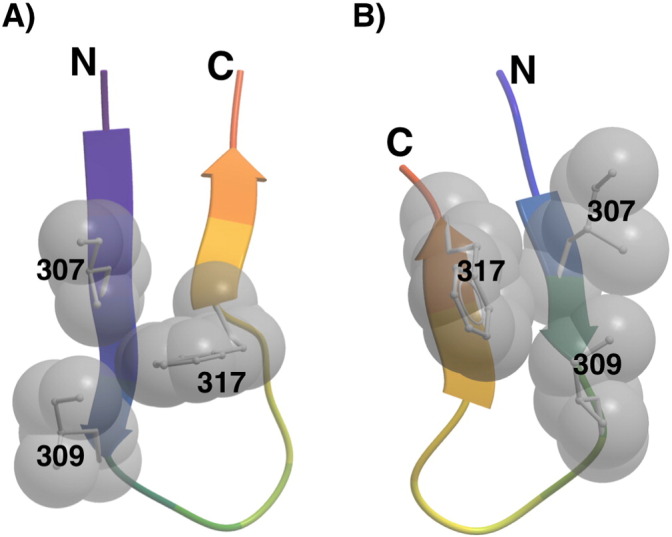
Structure of the V3 hydrophobic core. The side chains of V3 residues I^307^, I^309^, and F^317^ often pack against each other to form a hydrophobic core and are spatially located at either side of the hairpin turn in the V3 crown as represented by the V3 structures in complex with V3-specific mAb 447-52D (A) (Killikelly et al., 2013) or mAb 2557 (B) (Jiang et al., 2010). The V3 hairpin is drawn as a ribbon, and the side chains (toward the reader) of I^307^, I^309^ and F^317^ as sticks and CPK spheres.

V3 structures in complex with mAbs 447-52D (PDB ID 4M1D) and 2557 (PDB ID 3LMR) show that the V3 hydrophobic core can occur on either side of the V3 crown (Fig. 6, Jiang et al., 2010, Killikelly et al., 2013). For the amino acids identified in the sieve analysis at position 317 (Phe, Leu, Trp, Tyr, and Ile, Fig. 2B), all side chains of the V3 crown were energy-minimized by a Monte Carlo procedure using the ICM software package (Abagyan et al., 1997). The V3 hydrophobic core composed of residues 307, 309 and 317 was then analyzed for their side chain contact areas. In the 447-52D bound conformation, the residue 317 side chain contact areas in the core were calculated to be 12.4 Å^2^, 6.2 Å^2^, 16.8 Å^2^, 12.4 Å^2^, and 0.9 Å^2^, for amino acids Phe, Leu, Trp, Tyr, and Ile, respectively. Similarly for the case of mAb 2557 V3-bound conformation, the calculated side chain contact areas were 16.0 Å^2^, 2.9 Å^2^, 22.6 Å^2^, 17.1 Å^2^, 12.9 Å^2^, respectively. The results show that F^317^ provides the best measure for formation of the hydrophobic core in terms of side chain contact areas and hydrophobicity, except in the case of Trp. The bulky side chain of a Trp residue may be suboptimal for packing against V1V2 in the trimer (Lyumkis et al., 2013), and as noted above, the F317W mutation abrogates infectivity (Fig. 5). Notably, the two F317X breakthrough viruses in vaccinees (with W^317^ or L^317^) displayed rare mutations in the crown of V3 (Table 3) suggesting that for survival, viruses with F317X require extensive V3 mutations to simultaneously infect and escape Ab pressure. It is noteworthy that both of these two X^317^ vaccine breakthrough viruses retained the I^307^ residue. Moreover, in the just published 3.5 Å resolution structure of BG505 SOSIP.664 (Pancera, Zhou et al. 2014), the hydrophobic core of V3 has exactly the same conformation as that recognized by V3 mAb 2557, and it packs under several hydrophobic residues of V2. Thus the computational analysis reported above based on the 2557-bound V3 structure is consistent with the SOSIP conformation. Interestingly, V3 mAb 447-52D was shown to be able to bind the BG505 SOSIP.664 trimer (Pancera et al. 2014). This indicates that V3 can swing out from under V2, even in the stabilized SOSIP, and, in addition, the V3 hydrophobic core can flip from the conformation recognized by mAb 2557 to that recognized by mAb 447-52D (Fig. 6).

## 4 Discussion

The RV144 clinical trial showed an estimated VE of 31.2%, and that V2-directed IgG Abs were found to be a significant inverse correlate of infection risk. Recently it was suggested that Abs reactive with a linear V3 peptide from CRF01_AE also inversely correlated with infection risk in vaccinees who had low levels of Env-specific plasma IgA, ADCC and neutralizing Abs (Gottardo et al., 2013). Here we show that RV144 induced highly cross-clade reactive V3 Abs. In addition, sieve analysis suggests that breakthrough viruses differed between the vaccine and placebo groups at V3 positions 307 and 317. These analyses were supported by immunochemical studies showing RV144 plasma Abs were less reactive with cyclic V3 variants of HIV_B(BaL)_ and HIV_AE(A244)_ carrying substitutions for I^307^, and by virologic studies showing that substitutions at F^317^ decreased viral infectivity. Moreover, bioinformatics and structural analyses suggest that F^317^ is strongly favored at this position and appears to be optimal for the formation of the V3 hydrophobic core which is essential for maintaining the correct conformation of the V3 loop (Jiang et al., 2010). Taken together, the data suggest that vaccine-induced Abs that target I^307^ provided immune pressure and that, in the presence of vaccine-induced Abs, retention of F^317^ may confer an advantage to the virus, resulting in a VE of 85% against viruses with mutations at position 317 in V3.

The immunologic data demonstrate the broad cross-reactivity of vaccine-induced V3 Abs (Fig. 1 and Table 2) and recapitulate studies documenting the ability of many V3 mAbs to cross-react immunologically with diverse V3 peptides (Gorny et al., 1997), to capture virions from multiple subtypes (Nyambi et al., 2000), and to mediate cross-clade neutralization, though V3 mAbs preferentially neutralize sensitive Tier 1 viruses (Gorny et al., 1992, Conley et al., 1994, Corti et al., 2010, Hioe et al., 2010, Mouquet et al., 2011). Extensive structural data have demonstrated that glycan-independent human V3 mAbs target the mid-region (or “crown”) of the V3 loop (Stanfield et al., 2004, Burke et al., 2009, Gorny et al., 2009, Jiang et al., 2010). Sites 307 and 317 in the crown are both part of the hydrophobic core of the V3 epitope recognized by these mAbs. These residues are highly conserved, with I^307^ and F^317^ each present in eight of nine HIV subtype consensus sequences (Lynch et al., 2009), but whereas position 307 is a major contact residue, position 317 has minor or no contact with most V3-specific human mAbs (Stanfield et al., 2004, Stanfield et al., 2006, Burke et al., 2009, Jiang et al., 2010).

This extensive body of literature is augmented by studies of two mAbs, CH22 and CH23, which were isolated from vaccinees in the RV135 trial who received a vaccine regimen identical to that of RV144 (Montefiori et al., 2012). The epitopes recognized by these two mAbs are glycan-independent and react with V3 linear peptides in the crown of the V3 loop containing I^307^ and F^317^ (shown in bold and underlined: RKR**I**HIGPGRA**F**YTT and NTRTS**I**NIGPGQV**F**Y, respectively). Notably, these are not highly mutated Abs, having VH mutation frequencies of 3.7% and 4.5%, respectively, and each has a CDR H3 of 11 amino acids, characteristics similar to those found commonly in human Abs (Tiller et al., 2007). CH22 administered to macaques was able to protect against infection with SHIV BaL P4 (Haynes BF and Santra S, personal communication), extending similar work reported earlier (Watkins et al., 2011) and providing new data on the breadth of reactivity and the protective effects of V3 Abs. These data add support for the hypothesis that vaccine-induced V3-specific (and/or V2 specific) Abs contributed to the reduction in infection rate in vaccinees. While it is known that V3 Abs are induced early after infection and can drive rapid viral escape (Tomaras et al., 2008), the fact that the sieve analysis showed a difference between breakthrough viruses of vaccine and placebo recipients suggests that the V3 Abs exerted immune pressure in the vaccinees and therefore the vaccine-induced Abs had a biologic effect on the viruses that emerged in the setting of human infection.

The results of the RV144 study, identifying inverse correlates of risk related to levels of both V2 and V3 Abs (Haynes et al., 2012, Gottardo et al., 2013) recapitulate some of the many V2 and V3 commonalities. First, both crystallographic and cryo-EM studies of gp120 have shown that V2 and V3 are closely packed together at the apex of the envelope trimer (Bartesaghi et al., 2013, Julien et al., 2013, Lyumkis et al., 2013). Both V2 and V3 induce highly cross-reactive Abs in HIV-infected individuals (Gorny et al., 1997, Gorny et al., 2012); and V2 and V3 induce both glycan-independent (Gorny et al., 1997, Gorny et al., 2002) and glycan-dependent Abs (Pejchal et al., 2011). Most striking, perhaps, are the similarities revealed by the RV144 sieve analyses of V2, published earlier (Rolland et al., 2012), and the sieve analysis related to V3, described above. These studies suggest, independently, that immune pressure is exerted on viruses that match the vaccine at V2 position 169 and also at V3 position 307 (Fig. 2A). Vaccine efficacy for viruses matched with the vaccine at 169 is 48% (p = 0.004); VE for viruses matched with the vaccine at 307 is 52% (p = 0.004). The analogy continues to hold from the sieve analyses of site 181 in V2 and site 317 in V3 (Fig. 2B). For these latter positions, there was an atypical sieve effect with a VE of 78% (p = 0.003) against viruses with mutations at V2 site 181 (I181X), and a VE of 85% (p = 0.004) against viruses with mutations at V3 site 317 (F317X).

The implications of the sieve analyses along with immunologic and viral data suggest that both positions 169 (V2) and 307 (V3) are subject to immune pressure by RV144-induced Abs that target 169 (Liao et al., 2013, Zolla-Pazner et al., 2013) and 307 (XP Kong, personal communication and Montefiori et al., 2012). In contrast, the amino acid at 181 is either a poor or non-contact residue for RV144-induced V2-specific mAbs CH58 and CH59 or for mAbs that target the conformational “V2i” epitope (Liao et al., 2013, Mayr et al., 2013, Spurrier et al., 2014), and the amino acid at position 317 is a minor or non-contact residue for V3 mAbs (Stanfield et al., 2004, Burke et al., 2009, Jiang et al., 2010). The conservation of V2 site 181 and V3 site 317 and their preferential presence in vaccine breakthrough viruses leads to the hypothesis that these residues are not subject to direct immune pressure but instead, in the presence of the vaccine-induced Abs, must be retained in order to preserve the optimal structures of V2 and V3 that permit efficient infection (Jiang et al., 2010, Spurrier et al., 2014). It is noteworthy however, that statistical significance was not achieved for covariation between positions 307 and 317 within either the vaccine or placebo groups. In the vaccine group, 38 of 43 subjects were matched to both 181 and 317, and one subject was mismatched to both. In the placebo group, 40 of 65 subjects were matched to both 181 and 317, and 6 were mismatched to both (one placebo recipient subject was excluded for this analysis because of a sequencing artifact at site 317) (see Table S4).

The data relevant to immune pressure exerted by the immune response to the RV144 vaccine regimen have important implications for the design of an effective HIV vaccine. A 31% reduction in infection rate was achieved in RV144. The data support the hypothesis that broadly cross-clade reactive V3 and V2 Abs (Fig. 1 and Zolla-Pazner et al., 2014) played an important role in reducing the infection rate despite the fact that the vaccine-induced Abs display modest mutation rates from germline, i.e., < 5%, and none of the mAbs isolated to date from RV135 or RV144 vaccinees possess long CDR H3 regions (Montefiori et al., 2012, Liao et al., 2013). Similarly, Abs mediating ADCC also displayed modest levels of VH somatic mutation (0.5 to 1.5%) and showed considerable cross-clade activity (Bonsignori et al., 2012).

Although there was poor neutralizing Ab activity demonstrated in RV144 plasma (Montefiori et al., 2012), this must be interpreted cautiously since negative data in vitro do not prove that virus neutralization is not occurring in vivo. Thus, while a vaccine that reduces infection risk by only 31% surely needs improvement, the data available from RV144 suggest that vaccine-induced conventional Abs, i.e., those with modest rates of mutations in germline (< 5% VH mutation rates) (Montefiori et al., 2012, Liao et al., 2013) and without abnormally long CDR H3 regions or other structural anomalies, if targeted to the correct epitopes and present at appropriate levels over months to years should be effective (Zolla-Pazner, 2014). While desirable, broadly neutralizing, potent Abs that carry long CDR H3 regions and display extreme mutation rates from germline (as reviewed in Mascola and Haynes, 2013) have not yet been induced by any immunization regimen and, as demonstrated by RV144, are not necessarily needed to reduce infection risk. Therefore, the results of RV144 support a targeted effort to improve the design of immunogens such as those used in RV144, as well as recombinant epitope-scaffold immunogens to be used in prime-boost regimens that will induce conventional Abs now shown to be correlates of reduced risk of infection (Bonsignori et al., 2012, Haynes et al., 2012, Gottardo et al., 2013, Zolla-Pazner et al., 2014).

## 5 Conclusions

This study provides important new information supporting a critical role for vaccine-induced conventional antibodies in reducing the risk of HIV infection. In addition to immune pressure exerted by V2-specific antibodies, we show here that V3-specific antibodies also exerted immune pressure on breakthrough viruses infecting vaccinees in the RV144 vaccine trial. The results reveal the similarities in immune pressure exerted on the V2 and V3 regions of HIV-1 gp120 in vaccine recipients. These data inform the process of designing more effective candidate vaccines for reducing the rate of HIV infection.

## Conflict of Interest & Author Declaration

We wish to confirm that there are no known conflicts of interest associated with this publication and there has been no significant financial support for this work that could have influenced its outcome.

## Figures and Tables

**Table 1:**
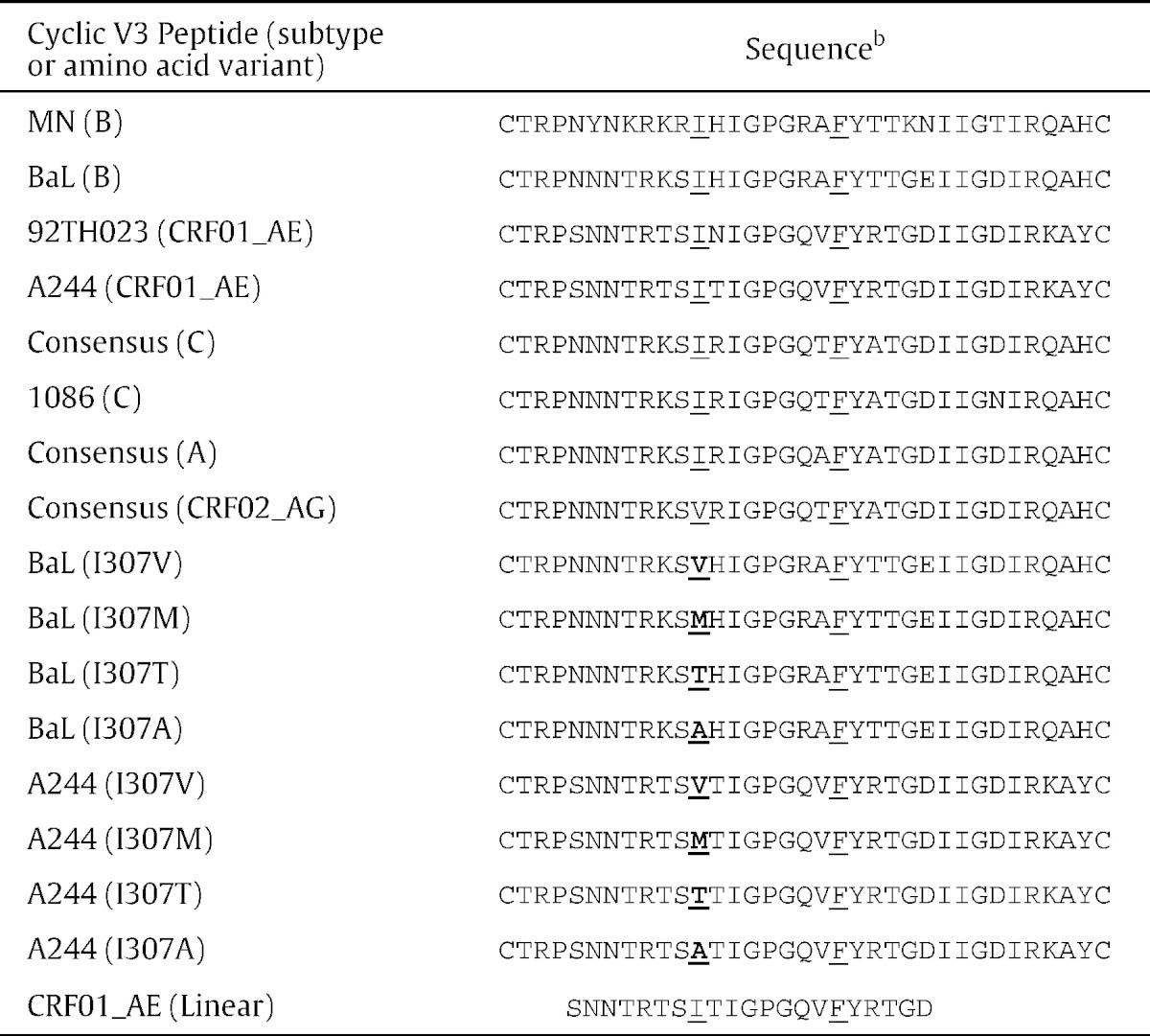
Sequences of cyclic V3 peptides used to analyze cross-reactivity and specificity of the RV144-induced antibody response directed against V3.^a^

^b^ Cyclic and linear peptides each have a biotin residue and a three glycine linker covalently bound to the N-terminal cysteine.

a Positions 307 and 317 are underlined and substitute amino acids are shown in bold.

**Table 2:**
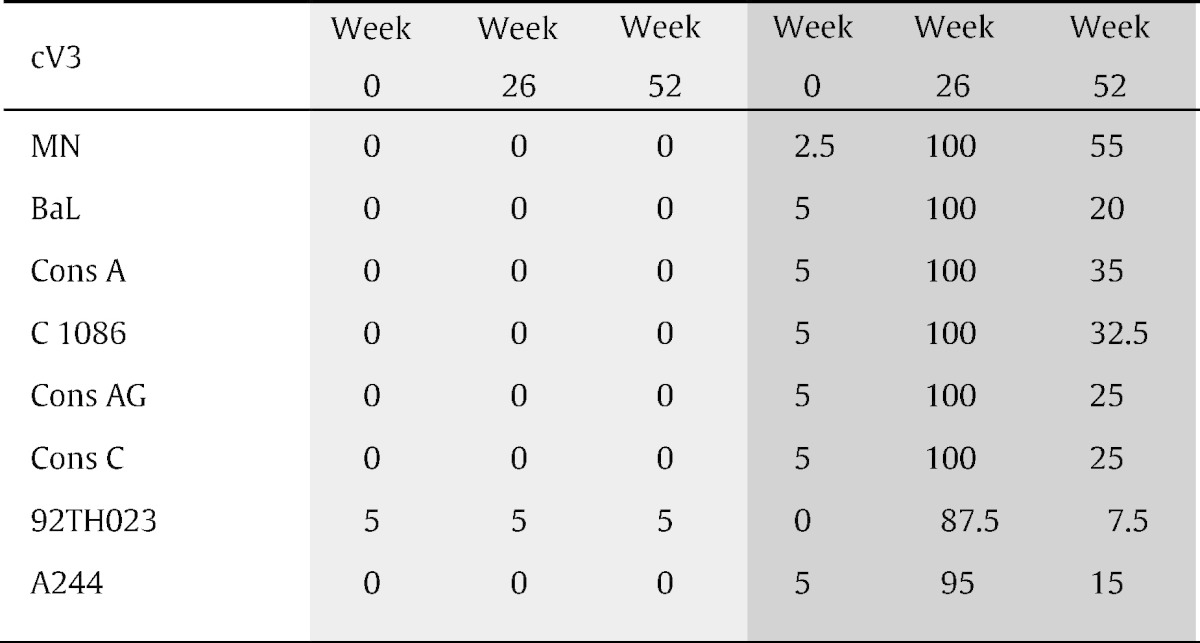
Longitudinal V3 antibody response rates in placebo and vaccine recipients.^a^

a Light gray = placebo recipients (n = 20); dark gray = vaccinees (n = 40). A positive response is defined as an optical density greater than a peptide-specific cut-off, calculated as three standard deviations above the mean of the week 0 readings.

**Table 3:**
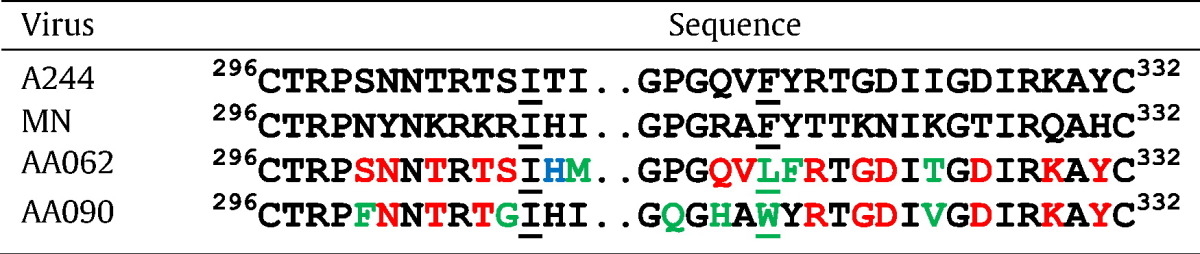
V3 sequences of gp120 immunogens and from the F317X breakthrough viruses found in two vaccine recipients.^a^

a V3 sequences with HxB2 numbering according to (Ratner et al., 1987). Underlined residues represent positions 307 and 317. Amino acids designated in blue differ from A244; red differ from MN; green differ from both the A244 and MN boosting protein immunogens.

**Table 4:** Comparison of amino acids in the boosting protein immunogens and those found in F317X breakthrough viruses from vaccinees.

Position #	Amino acid in boosting immunogens	Breakthrough virus designation	Amino acid mutation in breakthrough virus^a^	Frequency^b^
309	I	AA062	M	12.3%
317	F	AA062	L	4.0%
318	Y	AA062	F	2.8%
324	K&I	AA062	T	0.4%
300	N&S	AA090	F	10.5%
306	S&R	AA090	G	7.9%
313	P	AA090	Q	1.6%
315	R&Q	AA090	H	4.0%
317	F	AA090	W	2.4%
324	K&I	AA090	V	0%

a Refers to mutations shown in green in Table 3.

b Frequency is based on a curated dataset of CRF01_AE Env sequences derived from LANL (September, 2013) from which were removed those sequences that had out-of-frame mutations, stop codons, or were hypermutated.
