# Supplementary material for: Vaccine-induced Human Antibodies Specific for the Third Variable Region of HIV-1 gp120 Impose Immune Pressure on Infecting Viruses
Source: eBioMedicine. 2014 Nov 5;1(1):37–45. doi: 10.1016/j.ebiom.2014.10.022 (PMC4293639; doi:10.1016/j.ebiom.2014.10.022)
Supplement: Supplementary file 1 — Supplementary material. [file mmc1.doc]

**Supplementary Data**

for

**Vaccine-induced Human Antibodies Specific for the**

**Third Variable Region of HIV-1 Glycoprotein 120 Impose**

**Immune Pressure on Infecting Viruses**

by

Susan Zolla-Pazner^a,b^*, Paul T. Edlefsen^c^, Morgane Rolland^d^, Xiang-Peng Kong^b^, Allan deCamp^c^, Raphael Gottardo^c^, Constance Williams^b^, Sodsai Tovanabutra^d^, Sandra Sharpe-Cohen^b^ , James I. Mullins^e^, Mark S. deSouza^f^, Nicos Karasavvas^g^, Sorachai Nitayaphan^g^, Supachai Rerks-Ngarm^h^, Punnee Pitisuttihum^i^, Jaranit Kaewkungwal^i^, Robert J. O'Connell^g^, Merlin L. Robb^j^, Nelson L. Michael^j^,

Jerome H. Kim^j^, Peter Gilbert^c^

^^

**Supplementary Figure S1**. Spearman correlation coefficients are shown for vaccinees’ responses to cyclic V3 peptides at week 26. The heat map graphically displays the Spearman ranks with red depicting the highest correlations and yellow the weakest. Hierarchical clustering trees using complete linkage based on a distance measure defined as one minus the Spearman rank correlation coefficient between readouts were used to cluster the cyclic V3 peptides into common antigenic groups.

**Supplementary Table S1.**

**Pairwise Optical Density Difference in V3 Reactivity at Week 26***

| **Antigen** | |  |  |  |  |
| --- | --- | --- | --- | --- | --- |
|  |  | **Mean Diff.** | **Pct. Neg.** | **P-value** | **Adjusted P-value** |
| MN | A244 | 1.36 | 2.5 (1/40) | <0.00001 | <0.0001 |
| MN | 92TH023 | 1.32 | 2.5 (1/40) | <0.00001 | <0.0001 |
| MN | Con C | 1.21 | 0.0 (0/40) | <0.00001 | <0.0001 |
| MN | Con AG | 1.16 | 0.0 (0/40) | <0.00001 | <0.0001 |
| MN | C 1086 | 0.78 | 2.5 (1/40) | <0.00001 | <0.0001 |
| MN | Con A | 0.63 | 5.0 (2/40) | <0.00001 | <0.0001 |
| MN | BaL | 0.41 | 5.0 (2/40) | <0.00001 | <0.0001 |
| BaL | A244 | 0.95 | 7.5 (3/40) | <0.00001 | <0.0001 |
| BaL | 92TH023 | 0.91 | 7.5 (3/40) | <0.00001 | <0.0001 |
| BaL | Con C | 0.80 | 0.0 (0/40) | <0.00001 | <0.0001 |
| BaL | Con AG | 0.74 | 2.5 (1/40) | <0.00001 | <0.0001 |
| BaL | C 1086 | 0.36 | 12.5 (5/40) | <0.00001 | <0.0001 |
| BaL | Con A | 0.22 | 20.0 (8/40) | 3.3e-05 | 0.00023 |
| Con A | A244 | 0.73 | 0.0 (0/40) | <0.00001 | <0.0001 |
| Con A | 92TH023 | 0.69 | 5.0 (2/40) | <0.00001 | <0.0001 |
| Con A | Con C | 0.58 | 0.0 (0/40) | <0.00001 | <0.0001 |
| Con A | Con AG | 0.52 | 0.0 (0/40) | <0.00001 | <0.0001 |
| Con A | C 1086 | 0.14 | 10.0 (4/40) | <0.00001 | <0.0001 |
| C 1086 | A244 | 0.58 | 5.0 (2/40) | <0.00001 | <0.0001 |
| C 1086 | 92TH023 | 0.54 | 7.5 (3/40) | <0.00001 | <0.0001 |
| C 1086 | Con C | 0.44 | 0.0 (0/40) | <0.00001 | <0.0001 |
| C 1086 | Con AG | 0.38 | 0.0 (0/40) | <0.00001 | <0.0001 |
| Con AG | A244 | 0.21 | 30.0 (12/40) | 0.00038 | 0.0023 |
| Con AG | 92TH023 | 0.16 | 27.5 (11/40) | 0.0011 | 0.0053 |
| Con AG | Con C | 0.06 | 32.5 (13/40) | 0.0039 | 0.016 |
| Con C | A244 | 0.15 | 37.5 (15/40) | 0.029 | 0.086 |
| Con C | 92TH023 | 0.11 | 45.0 (18/40) | 0.082 | 0.16 |
| 92TH023 | A244 | 0.04 | 30.0 (12/40) | 0.08 | 0.16 |

*Difference in pairwise OD values between the first antigen minus the second antigen. Mean difference is shown followed by the percentage of negative differences along with the p-value from t-test (testing a null hypothesis that the true mean difference is zero). Adjusted P-values are based on the Holm FWER adjustment procedure

**Table S2.**

**Estimated Vaccine Efficacies to Prevent Infection with**

**Specific CRF01_AE HIV-1 Genotypes**

| **CRF01_AE** | **Numbers of Infections** | | **Vaccine Efficacy**^1^  **(95% CI)** | **2-sided** |
| --- | --- | --- | --- | --- |
| **HIV-1 Genotype** | **Vaccine** | **Placebo** |  | **P-value** |
| **(All)** | **43** | **66** | **35% (5%, 56%)** | **0.026** |
| I307 | **21** | **44** | **52% (20%, 72%)** | **0.004** |
| I307X | 22 | 22 | 0% (-44%, 45%) | 0.99 |
| F317 | 41 | 53 | 23% (-14%, 49%) | 0.21 |
| F317X | **2** | **13** | **85% (33%, 97%)** | **0.004** |
| I307-F317 | **19** | **38** | **50% (14%, 71%)** | **0.011** |
| I307-F317X | 2 | 6 | 67% (-39%, 93%) | 0.16 |
| I307X-F317 | 22 | 15 | -31% (-64%, 24%) | 0.26 |
| I307X-F317X | **0** | **7** | **100% (31%, 100%)** | **0.016** |

^1^ Symmetrized estimated vaccine efficacies (for positive HR, symmetrized VE = [1 – hazard ratio (HR)]×100%; for negative HR, symmetrized VE = – [1 – (1/(HR))]×100%) to prevent infection with specific HIV-1 genotypes, and estimated ratios of HRs measuring the relative protection against pairs of HIV-1 genotypes.

**Table S3.**

**Estimated Ratios of Genotype-specific Hazard Ratios**

|  | **Estimated HR/HR^1^**  **(95% CI)** | **P*-*value** |
| --- | --- | --- |
| I307X/I307 | **2.10 (0.95, 4.60)** | **0.065** |
| F317/F317X | **5.03 (1.07, 23.53)** | **0.040** |
| Else/I307-F317 | 1.71 (0.79, 3.72) | 0.17 |
| Else/I307-F317X | 2.05 (0.39, 10.68) | 0.39 |
| I307X-F317/Else | **3.56 (1.55, 8.17)** | **0.003** |
| Else/I307X-F317X | 2.00 (0.20, 19.88) | 0.55 |

^1^ Each HR is the hazard ratio (vaccine vs. placebo) of HIV-1 infections with a particular genotype. For example, for the F317/F317X entry, the numerator HR measures the vaccine effect to prevent HIV-1 infections with F317 and the denominator HR measures the vaccine effect to prevent HIV-1 infections with F317X-variants, and the result, 5.03, means that the vaccine lowers the rate of infection 5.03-times more against F317-mismatched HIV-1 than against F317-matched HIV-1. Else is a term for all genotypes other than the joint genotype under consideration. Values in bold are statistically significant

**Table S4.**

**Enumeration of Breakthrough Viruses Matched and Mismatched**

**at Positions 181 (in V2) and 317 (in V3)**

| **Treatment Group** |  | **Env 317** | **Env F317X** |
| --- | --- | --- | --- |
| Vaccine | **Env 181** | 38 | 1 |
|  | **Env 181X** | 3 | 1 |
| Placebo* | **Env 181** | 40 | 7 |
|  | **Env 181X** | 12 | 6 |

*One subject was excluded because of a sequencing artifact at 317
